# Polyethylene glycol-coated ultrasmall superparamagnetic iron oxide nanoparticles-coupled sialyl Lewis X nanotheranostic platform for nasopharyngeal carcinoma imaging and photothermal therapy

**DOI:** 10.1186/s12951-021-00918-0

**Published:** 2021-06-08

**Authors:** Qinmin Liu, Lijuan Liu, Chunwei Mo, Xiao Zhou, Dongming Chen, Yu He, Hailu He, Wei Kang, Yongfeng Zhao, Guanqiao Jin

**Affiliations:** 1 Department of Radiology, Guangxi Medical University Cancer Hospital, Nanning, 530021 China; 2 Department of Chemistry and Biochemistry, Jackson State University, Jackson, MS 39217 USA

**Keywords:** Nasopharyngeal carcinoma, Nanotheranostic platform, USPIO-PEG-sLe^x^, Magnetic resonance imaging, Photothermal therapy

## Abstract

**Background:**

Nasopharyngeal carcinoma (NPC) is a type of head and neck malignant tumor with a high incidence in specific regional distribution, and its traditional therapies face some challenges. It has become an urgent need to seek new therapeutic strategies without or with low toxicity and side effects. At present, more and more researchers has been attracting attention by nanotheranostic platform. Therefore, our team synthesized the polyethylene glycol-coated ultrasmall superparamagnetic iron oxide nanoparticles-coupled sialyl Lewis X (USPIO-PEG-sLe^x^) nanotheranostic platform with high temperature pyrolysis.

**Results:**

The USPIO-PEG-sLe^x^ nanoparticles had excellent photothermal conversion property, and the temperature of USPIO-PEG-sLe^x^ nanoparticles solution increased with its concentration and power density of near-infrared (NIR) on 808 nm wavelengths. Five USPIO-PEG-sLe^x^ nanoparticles with different concentrations of 0 mg/ml, 0.025 mg/ml, 0.05 mg/ml, 0.1 mg/ml and 0.2 mg/ml were prepared. The biological toxicity results showed that the viability of NPC 5-8F cells is related to the concentration of USPIO-PEG-sLe^x^ nanoparticles and the culture time (P < 0.001). The results of photothermal therapy (PTT) in vitro indicated that the viability of 5-8F cells decreased significantly with the concentration of USPIO-PEG-sLe^x^ nanoparticles increases (P < 0.001), and the viability of NPC 5-8F cells were 91.04% ± 5.20%, 77.83% ± 3.01%, 73.48% ± 5.55%, 59.50% ± 10.98%, 17.11% ± 3.14%, respectively. The USPIO-PEG-sLe^x^ nanoparticles could target the tumor area, and reduce the T2* value of tumor tissue. The T2* values of tumor pre- and post-injection were 30.870 ± 5.604 and 18.335 ± 4.351, respectively (P < 0.001). In addition, USPIO-PEG-sLe^x^ nanoparticles as a photothermal agent for PTT could effectively inhibit tumor progression. The ratio of volume change between tail vein injection group, control group, nanoparticles without laser irradiation group and blank group after 5 treatments were 3.04 ± 0.57, 5.80 ± 1.06, 8.09 ± 1.96, 7.89 ± 2.20, respectively (P < 0.001).

**Conclusions:**

Our synthesized USPIO-PEG-sLe^x^ nanotheranostic platform, and it may be become a new strategy for the treatment of NPC.

**Graphic Abstract:**

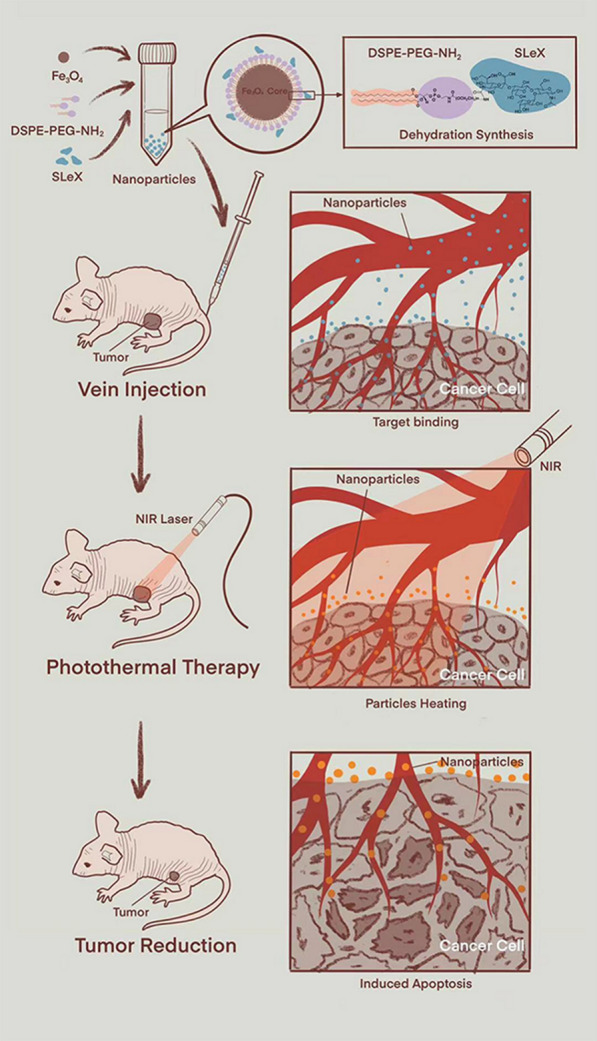

## Background

Nasopharyngeal carcinoma (NPC) is a type of head and neck malignant tumor with a high incidence in specific regional distribution. According to data in 2018 released by the International Agency for Research on Cancer (IARC), there are 129,079 new cases of NPC in worldwide [1], and 84.6% of the cases are in Asian countries, and the top three age-standardized rates are in Brunei, Penang, Malaysia and China [2]. In China, the vast majority of NPC occurs in five southern provinces, Guangdong, Guangxi, Hainan, Hunan, and Fujian [3]. The development of NPC is related to many factors, such as Epstein-Barr virus, environment, genetics [4], etc. Epstein-Barr virus, an independent risk factor, is closely related to the development and progression of NPC, especially undifferentiated NPC [5]. In the past few decades, the treatment strategies for NPC have been dominated by radiotherapy, chemotherapy or combination therapy [4]. However, these methods have some disadvantages. Radiation resistance has not only cause local progression, but also leads to a higher mortality [6]. Chemotherapy can easily bring systemic side effects to patients, and even death [7]. In emerging Immunotherapy, the different sensitivity of patients to Immunotherapy and combined with other treatment methods is still in clinical trials, and the existing immune resistance has become a bottle-neck in the application of Immunotherapy in NPC [8, 9]. Therefore, seeking a non-invasive, low-toxic, and efficient treatment strategy for NPC has become an urgent clinical need. The nanotheranostic platform which is a Nanocarrier that integrates diagnosis and treatment, has been favored by the researchers [10]. Most of nanotheranostic platforms are nanocomposites with imaging function nanoparticles coupled to therapeutic agents. For example, Abed et al. synthesized Iron (III) oxide–gold (Fe2O3@Au) as a core–shell nanoparticles platform is used for magnetic resonance imaging (MRI) T2 sequence and photothermal therapy (PTT) [11]. Zhang et al. prepared Multi-walled carbon nanotubes-magnetofluorescent carbon dots/doxorubicin (GdN@C quantumQDs-MWCNTs) nanocomposites applied for MRI T1 sequence, and can be used for PTT and chemotherapy [12].

**Fig. 1:**
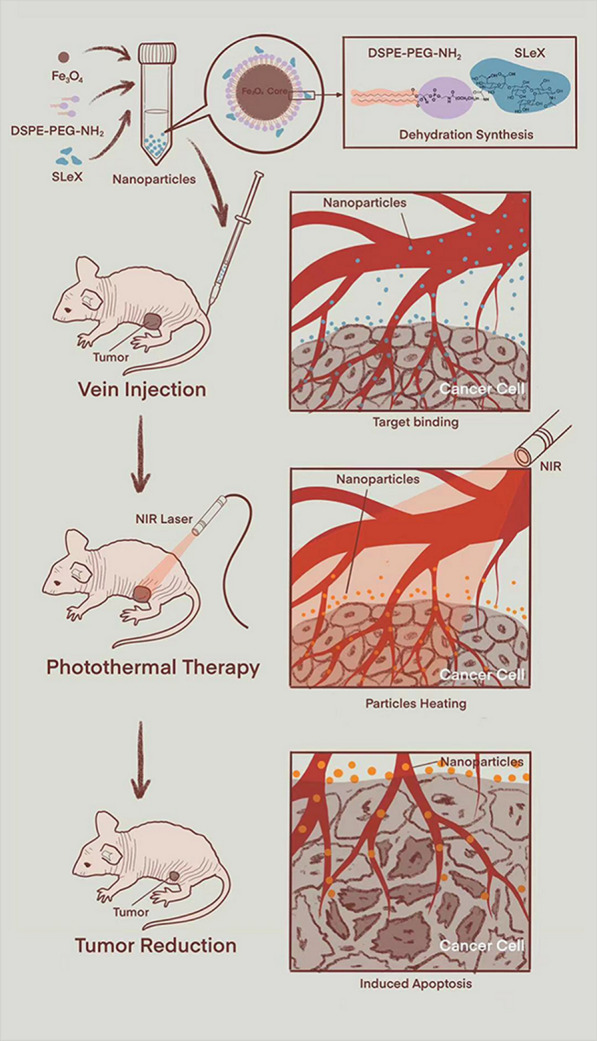
Schematic diagram of PTT (using animal experimental research as a model)

PTT emerged in the 1980’s and has developed rapidly in the recent years. The basic principle of PTT is that a photothermal agent (PA) is absorbed light radiation, such as near-infrared (NIR) light, and generate heat energy so as to ablate tumor cells and regress the tumor (Fig. 1) [13]. Compared with traditional tumor treatment methods such as surgery, radiotherapy, chemotherapy, etc., PTT has many advantages like non-invasiveness, high specificity, high selectivity and controllability [13, 14]. Now, most PAs of PTT are nanomaterials, which are divided into inorganic nanomaterials and organic nanomaterials [15]. Inorganic nanomaterial’s mainly include metal nanomaterials, carbon-based nanomaterials, etc. [16, 17]. Some metal nanomaterials approved by the American Food and Drug Administration (FDA) as a contrast agent for the MRI in clinical, like ultrasmall superparamagnetic iron oxide (USPIO). USPIO nanoparticles can significantly increase the transverse relaxation rate of MRI, shorten the T2 value [18], and have no obvious biological toxicity [19]. In addition, due to the outstanding stability, excellent biocompatibility and photothermal conversion efficiency of USPIO nanoparticles, many researchers also explore USPIO nanoparticles or their chelates as PAs [11, 20]. Now, nano-PAs has gotten extensive attention from more and more researchers as a nanotheranostic platform.

**Fig. 2:**
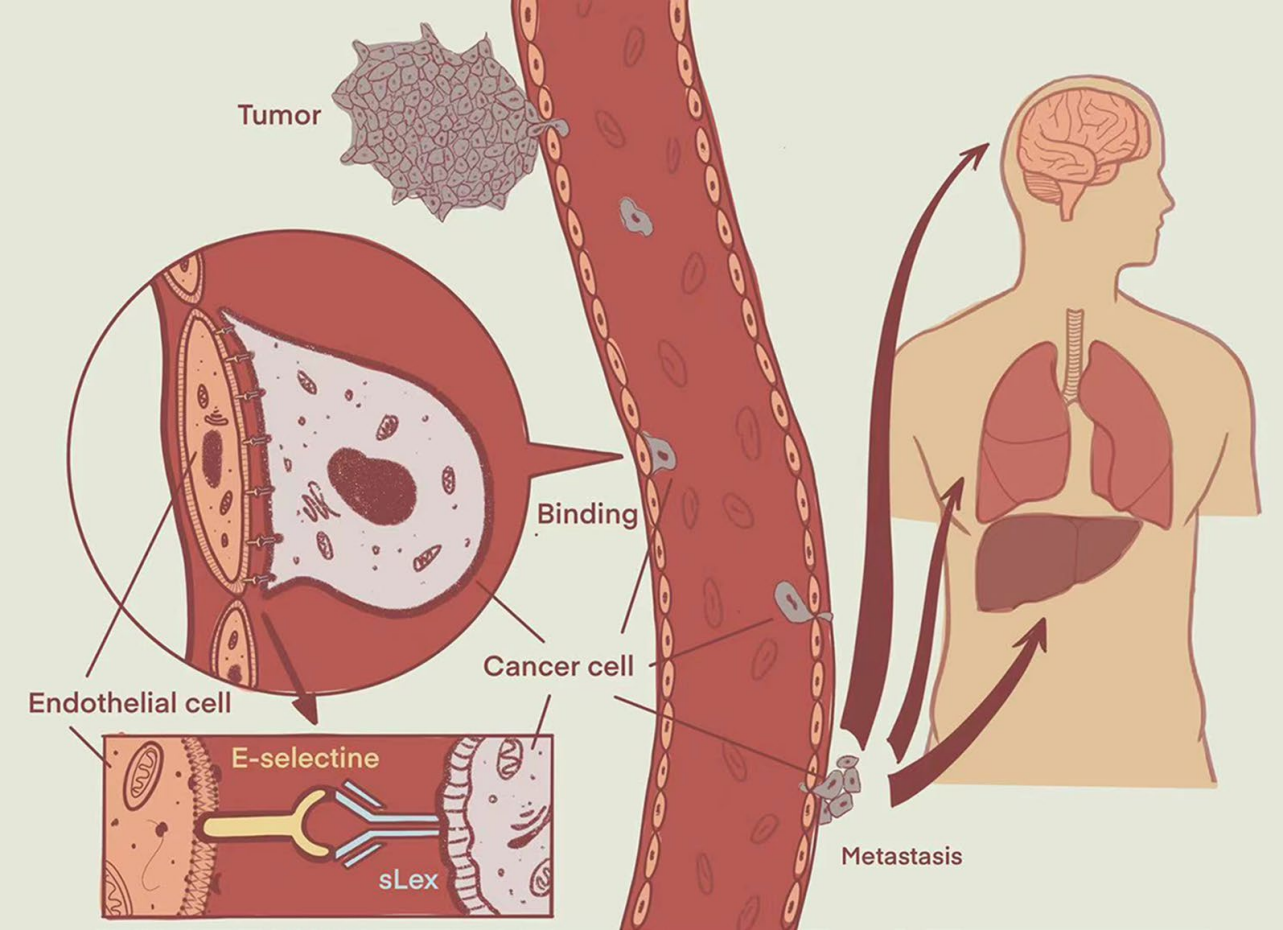
Schematic diagram of the binding between sLe^x^ and E-selectin

E-selectin is a kind of transmembrane protein, also known as CD62E, ELAM-1 and LECAM-2. Its primary structure consists of a C-type lectin-like domain, an epidermal growth factor-like domain and a complement-like domain, which has six repeats (about 60 amino acids per sequence) [21, 22]. E-selectin often expressed in the vascular endothelial cells of inflammation or cancer [21, 22]. Studies have shown that E-selectin related to the progression, metastasis and prognosis of a variety of malignant tumors, such as breast cancer [23], gastric cancer [24], and NPC [25]. The plasma membrane of cancer cells can also express the specific ligand of E-selectin, namely sialyl Lewis X (sLe^x^) (Fig. 2) [26], which might indicate that E-selectin is a natural target for anti-cancer therapy.

Therefore, in our study, our team synthesized polyethylene glycol (PEG)-coated USPIO nanoparticles-coupled sLe^x^ (USPIO-PEG-sLe^x^) nanocomplex (Fig. 1), which had an outstanding dispersion, high stability, excellent T2 relaxation, and could targrt the E-selectin expression of tumor neovascular endothelial cells, and the USPIO-PEG-sLe^x^ were performed for MRI and PTT of NPC nude mouse xenograft models as a nanotheranostic platform.

## Materials and methods

### Materials

Iron(III)2,4-pentanedionate, 1,2-hexadecanediol, oleic acid, oleylamine, EDC solid powder, phenyl ether and MES buffer solution were obtained from Shanghai Aladdin Company. Ethyl alcohol, hexyl hydride and trichloromethane were abtained from Shanghai Sinopharmm Group Chemical Reagent Co. LTD. DSPE-PEG2000 solid powder (A.V.T., Shanghai, China), sLe^x^ (Carbosynth, UK), 808 nm near-infrared laser (BWT, Beijing, China), Digital thermometer (TES, Taipei, China), Cell Counting Kit-8 (Dojindo, Shanghai, China), Trypan blue staining kit (Solarbio, Beijing, China), 3.0T MRI scanner (Discovery MR750, GE Healthcare, USA), four-channel animal coil (10F-04885, Teshen, Shenzhen, China), Near-infrared thermal imager (HIKVISION, Hangzhou, China), Prussian blue iron stain kit (Biotopped, Beijing, China), Hematoxylin–eosin stain kit (Solarbio, Beijing, China), Anti-CD62E antibody (Abcam, Shanghai, China).

### Synthesis of USPIO-PEG-sLe^x^

2 mmol iron(III)2,4-pentanedionate, 10 mmol 1,2-hexadecanediol, 20 ml phenyl ether, 6 mmol oleic acid and 6 mmol oleylamine were heated to 200 °C for 1 h, then heated to 256 °C for half an hour. The solution cooled to room temperature, and then added 40 ml ethyl alcohol into the reaction solution. Centrifugation was conducted for 10 min at 6000 r/min to obtain black sediments. The process of ethanol precipitation/non-polar solvent dispersion was repeated 2–3 times to obtain the ferroferric oxide nanoparticles which with an average size of 6 nm and coated with oleic acid. The 84 mg ferroferric oxide nanoparticles synthesized by the above method were dispersed in 4 mL hexyl hydride solution and then added with 2 mmol iron(III)2,4-pentanedionate, 10 mmol 1,2-hexadecanediol, 20 ml phenyl ether, 6 mmol oleic acid and 6 mmol oleylamine to finally obtain the ferroferric oxide nanoparticles with an average size of 10 nm. Took 50 mg distearoyl phosphoethanolamine-PEG2000 (DSPE-PEG2000) solid powder dissolved in 5 ml trichloromethane, then added 5 ml ferroferric oxide nanoparticles solution. The mixed solution moved to 50 ml round bottom flask and fully shocked 10 min via ultrasonic apparatus under 70 °C, then 5 ml deionized water was added. The flask was placed on a rotary evaporator, the water bath was 70 °C, and the flask was pumped to vacuum before rotary evaporation. The samples were filtered by 220 nm filter membrane and then ultrafiltration centrifuged to remove the bottom sediment. USPIO-PEG nanoparticles were obtained by taking the solution with black and transparent aqueous phase nanostructure on the upper layer.

Dissolved 2 mg sLe^x^ in 2 ml pure water to obtain 1 mg/ml sLe^x^ solution. 1.1 ml MES (0.15 mol/l, pH 5.5) buffer solution was added into 11 ml USPIO-PEG nanoparticles and 120 ul MES (0.15 mol/l, pH 5.5) buffer solution was added into 1.2 ml sLe^x^ solution (1 mg/ml) to adjust the pH value. Then, the USPIO-PEG nanoparticles solution swirled into the sLe^x^ solution and incubated for 30 min at 37 °C in a shaker, and 300 ul 1-(3-Dimethylaminopropyl)-3-ethylcarbodiimide hydrochloride (EDC) solution with a solubility of 10 mg/ ml was added into the mixed solution and incubated at 37 °C overnight. Then, the mixed solution was ultrafiltration for four times by 100KD ultrafiltration tube and pure water, and the excess EDC and unreacted sLe^x^ were washed. After ultrafiltration, the suspension was diluted to 8 ml with pure water, and the iron concentration was determined by phenanthroline spectrophotometric determination. Then, the concentration of USPIO-PEG-sLe^x^ nanoparticles solution with iron concentration of 1 mg/ml was obtained.

### Characterization of USPIO-PEG-sLe^x^

The size, shape, and dispersion of USPIO-PEG-sLe^x^ were measured by transmission electron microscopy (TEM, JEM-2100, Japan). The hydrodynamic size and Zeta potential of the nanoparticles were measuured by dynamic light scattering (DLS) using the Zetasizer Nano ZS90 (Malven, UK). The Fourier Transform Infrared (FTIR) spectra was measured with the fourier infrared spectrometer (Thermo Nicolet, USA).

### Photothermal property test of USPIO-PEG-sLe^x^ in vitro

Dilute the USPIO-PEG-sLe^x^ nanoparticles solution with the pure water to five concentrations of 0 mg/ml, 0.025 mg/ml, 0.05 mg/ml, 0.1 mg/ml and 0.2 mg/ml. 1 ml of each concentration placed into a glass colorimetry cup, and then a near-infrared laser with a wavelength of 808 nm to irradiate 5 different concentrations of USPIO-PEG-sLe^x^. The power densities are 0.7 W/cm^2^, 1.4 W/cm^2^, and 2.1 W/cm^2^ for 10 min, respectively. Recorded the temperature changes of different concentrations of USPIO-PEG-sLe^x^ solution every 10 s.

### Cytotoxicity test of USPIO-PEG-sLe^x^

Took 5-8F cells, during the logarithmic growth phase, they were planted in four 96-well plates, at a cell concentration of 5 × 10^4^/ml, inoculated 100μL of cell suspension per well, and then placed them into a constant temperature incubator at 37 °C and 5% CO_2_ concentration culture for 24 h, and then took out and changed the medium. Each of the four experimental groups has 6 replicate wells. 10µL of the concentrations of USPIO-PEG-sLe^x^ solution which included 0.025 mg/ml, 0.05 mg/ml, 0.1 mg/ml, and 0.2 mg/ml was added to each well. In addition, set up a control group (without USPIO-PEG-sLe^x^ solution) and a blank group (without cells and USPIO-PEG-sLex solution). The 96-well plates placed into a constant temperature incubator to culture for 2, 4, 8, and 24 h, respectively. Then took the 96-well plates out, discard the medium, and rinse three times with PBS buffer solution, then added 100µL of fresh medium and 10µL of CCK8 reagent to each well of each group.The 96-well plates then placed into constant temperature in the incubator for 2 h. The absorbance at the wavelength of 450 nm recorded by a microplate reader. The cell viability is calculated by the following formula:

$${\text{Cell viability}} = \left[ {\left( {{\text{As}} - {\text{Ab}}} \right)/\left( {{\text{Ac}} - {\text{Ab}}} \right)} \right] \times {1}00\%$$

the As is the absorbance of the experimental well, Ac is the absorbance of the control well, Ab is the absorbance of the blank well.

### PTT of USPIO-PEG-sLe^x^ in vitro

Took 5-8F cells in logarithmic growth phase, planted them in a 96-well plate at a cell concentration of 5 × 10^4^/ml. Inoculated 100 μL of cell suspension per well, and then placed the plate in a 37 °C constant temperature incubator, 5%CO_2_ concentration for 24 h. Each of the five experimental groups has 6 replicate holes. Five concentrations of USPIO-PEG-sLe^x^ solution of 0 mg/ml, 0.025 mg/ml, 0.05 mg/ml, 0.1 mg/ml, and 0.2 mg/ml to replace the medium in the well. Each well then irradiated with 1.4 W/cm^2^ NIR power density laser at 808 nm wavelength for 10 min. Then, all wells were washed three times with PBS buffer. In addition, the control group (no USPIO-PEG-sLe^x^ solution, no NIR irradiation) and a blank group (only complete medium, no NIR irradiation) set up. Three groups respectively added with 100 μL of complete medium and 10 μL of CCK8 reagent to each well. The plate placed in a constant temperature incubator at 37 °C, 5% CO_2_ concentration for 2 h. The cell viability described by the above formula. Dead cells stained in the experimental group with 0.4% trypan blue staining solution.

### MRI of USPIO-PEG-sLe^x^ and USPIO-PEG, Prussian blue iron staining, and immunohistochemical (IHC) staining

The MRI sequences included coronal T2WI, transverse T1WI, T2WI, and T2*map.

USPIO-PEG-sLe^x^ nanoparticle solutions with concentrations of 0 mg/ml, 0.025 mg/ml, 0.05 mg/ml, 0.1 mg/ml, and 0.2 mg/ml installed in EP tubes in vitro MRI. After scanning, the T2*map of different concentrations of USPIO-PEG-sLe^x^ nanoparticles was drawn through the MRI post-processing workstation.

BALB/C nude mice, purchased from the Experimental Animal Center of Guangxi Medical University (production license number is SCXK GUI 2014-0002, application license number is SYXK GUI 2014-003). 5-8F, configure the cell concentration to 1 × 10^7^/ml with saline, and then configure it to 0.1 ml of 5-8F cell suspension, and then planted it subcutaneously on the right ventral side of each nude mouse. MRI examination was performed after 2 weeks. Twelve nude mice were randomly divided into USPIO-PEG-sLe^x^ group and USPIO-PEG group. The USPIO-PEG-sLe^x^ and USPIO-PEG nanoparticles were injected into the two group mice via the tail vein, respectively. Each group of nude mice were underwent MRI twice. The first MRI performed by the pre-injection of nanoparticles. One hour after injecting 0.1 ml of nanoparticles through the tail vein, MRI was performed again. The T2* values of the tumors measured by the magnetic resonance post-processing workstation, and then differences in the T2* values pre- and post-injection of nanoparticles in each group analyzed. After the second MRI, nude mice are then sacrificed by the cervical spinal dislocation, and their tumors, brains, hearts, livers, kidneys, and spleens then collected. All tissues were fixed with 10% formalin.

Subsequently, all tissue specimens of the two groups were dehydrated and embedded in paraffin. Paraffin blocks were cut into 4 μm thick sections. Prussian blue iron stain kit was used for iron staining. The positive area percentage of iron staining in different tissue was analyzed between the two groups. The expression of E-selectin in tumor specimens was analyzed by IHC staining, and differences of E-selectin in the USPIO-PEG-sLe^x^ group and USPIO-PEG group were analyzed using mean optical density (MOD) by ImageJ measurement.

### PTT of USPIO-PEG-sLe^x^ in vivo, HE staining of tumor

24 tumor-burdened nude mice randomly divided into four groups: tail vein injection group, control group, nanoparticles without laser irradiation group and blank group. Nude mice in the tail vein injection group injected with 0.1 ml of USPIO-PEG-sLe^x^ nanoparticle via the tail vein for 1 h and then received PTT. The control group was performed PTT after injection of saline. The two groups irradiated for 10 min with a power density of 0.7 W/cm^2^. The nanoparticles without laser irradiation group was injected with 0.1 ml of USPIO-PEG-sLe^x^ nanoparticle via tail vein without PTT. The blank group received neither USPIO-PEG-sLe^x^ nanoparticles nor PTT. During the treatment, a NIR thermal imaging device used to monitor the temperature change of the tumor in real time. There are 72 h between each treatment and a total of 5 treatments. The body weight and tumor size of nude mice were measured before and every three days after treatment. The tumor size calculated according to the following formula:

$${\text{V}} = {\text{a}} \times {\text{b}}^{{2}} /{2}$$

There, a is the long diameter of the tumor and b is the short diameter of the tumor. After the five times’ treatment, the four groups of nude mice sacrificed by cervical spinal dislocation to obtain tumors, and the tumors were fixed with 10% formalin.

Tumor specimens of nude mice in all groups were dehydrated and embedded in paraffin. The paraffin block was cut into 4 μm thick sections, then the tumor specimens of each group of nude mice were stained with HE. The tumor cell apoptosis observed under the microscope via HE staining.

### Statistical analysis

SPSS 23.0 statistical software was used for data analysis. The data of each group were compiled by mean ± standard deviation $\left( {{\overline{\text{x}}} \pm {\text{s}}} \right)$. The differences were analyzed by paired t test, independent-samples t test, analysis of variance (ANOVA) and Chi-square test. Differences between different groups was compared by LSD-t test. Take P ≤ 0.05, because the difference is statistically significant.

## Results

### Characterization of composition, hydrodynamic size, zeta potential, and fourier transform infrared spectroscopy

**Fig. 3:**
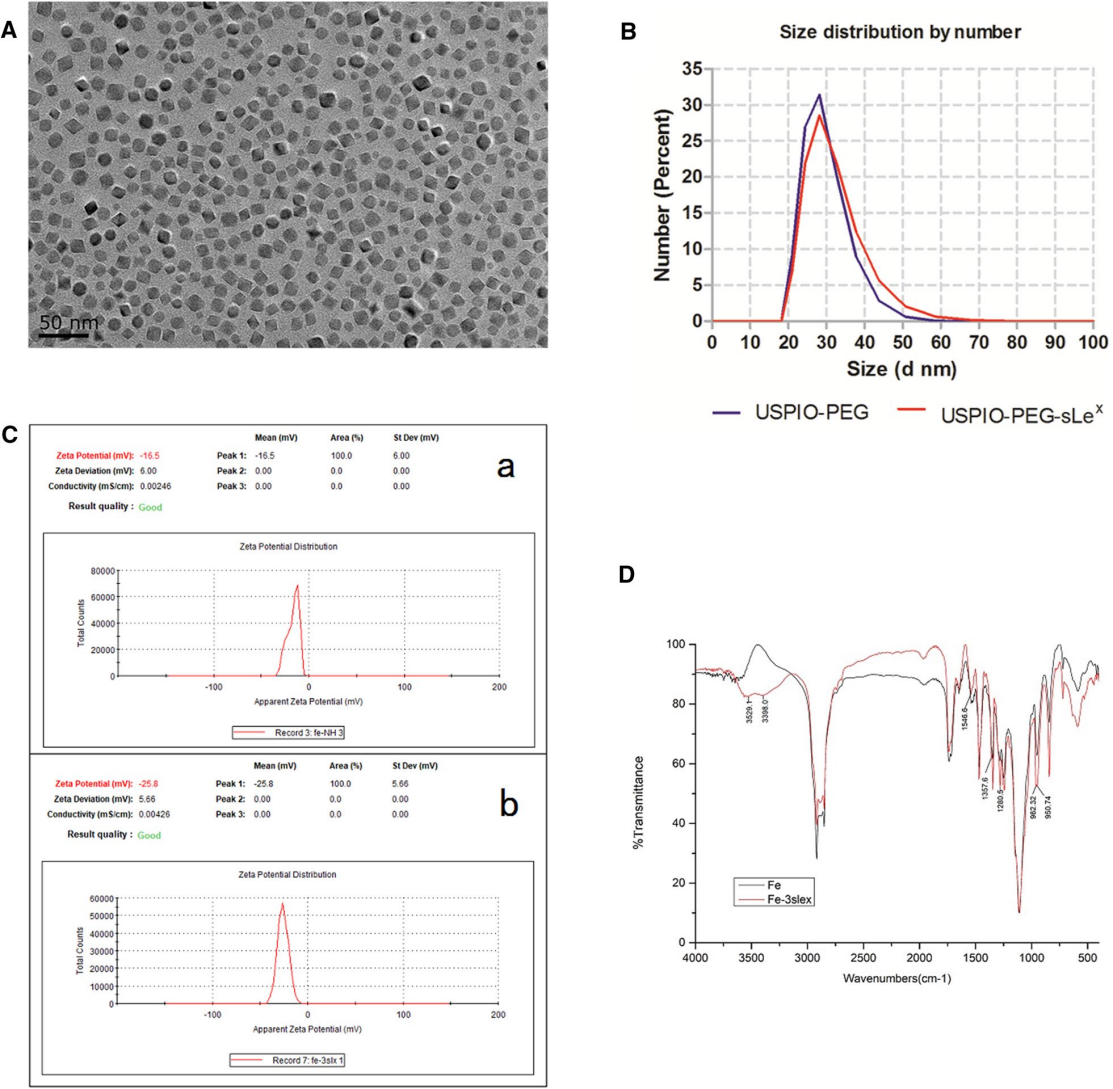
Characterization of of USPIO-PEG-sLe^x^ nanoparticles. **A** TEM of USPIO-PEG-sLe^x^. Most of the USPIO-PEG-sLe^x^ nanoparticles were square and polygonal, while a few were spherical. **B** The number size of USPIO-PEG and USPIO-PEG-sLe^x^. **C** The Zeta potential of USPIO-PEG (a) and USPIO-PEG-sLe^x^ (b). **D** Fourier Transform Infrared (FTIR) spectra of USPIO-PEG and USPIO-PEG-sLe^x^

TEM showed that most of the USPIO-PEG-sLe^x^ nanoparticles were square and polygonal, while a few were spherical (Fig. 3A). The dispersion degree was favorable, and the size of USPIO-PEG-sLe^x^ was about 10 nm which was relatively uniform. The DLS showed that the number size of USPIO-PEG and USPIO-PEG-sLe^x^ were 28.9 nm ± 5.596 nm and 30.65 nm ± 7.061 nm, respectively (Fig. 3B). This indicated that the distribution of nanoparticles size was narrow. The Zeta potential of USPIO-PEG and USPIO-PEG-sLe^x^ were − 16.5 ± 6 mV and − 25.8 ± 5.66 mV, respectively (Fig. 3C). Owe to the presence of phospholipid groups in DSPE-PEG-NH_2_, USPIO-PEG had negative charge on its surface. After coupling with sLe^x^, the Zeta potential of USPIO-PEG-sLe^x^ showed a larger negative charge. It may be because the amino groups on the surface of USPIP-PEG were occupied by sLe^x^. The coupling between USPIO-PEG and sLe^x^ was proved to be successful via hydrodynamic size and Zeta potential detection.

FTIR spectra (Fig. 3D) showed that double peaks appeared at 3529.1 cm^−1^ and 3398.0 cm^−1^, which was the stretching vibration peak of primary amide N–H after coupling, and the stretching vibration peak of amide C–N appeared at 1357.6 cm^−1^. The single peak at 960 cm^−1^ became a double peak of 962.32 cm^−1^ and 950.74 cm^−1^ owe to rolling vibration of methyl groups at the end of the pyran ring. The changes of pre- and post-coupling could been observed via the infrared spectrum, which indicated that USPIO-PEG and sLe^x^ were successfully coupled.

### Photothermal property of USPIO-PEG-sLe^x^ in vitro

**Fig. 4:**
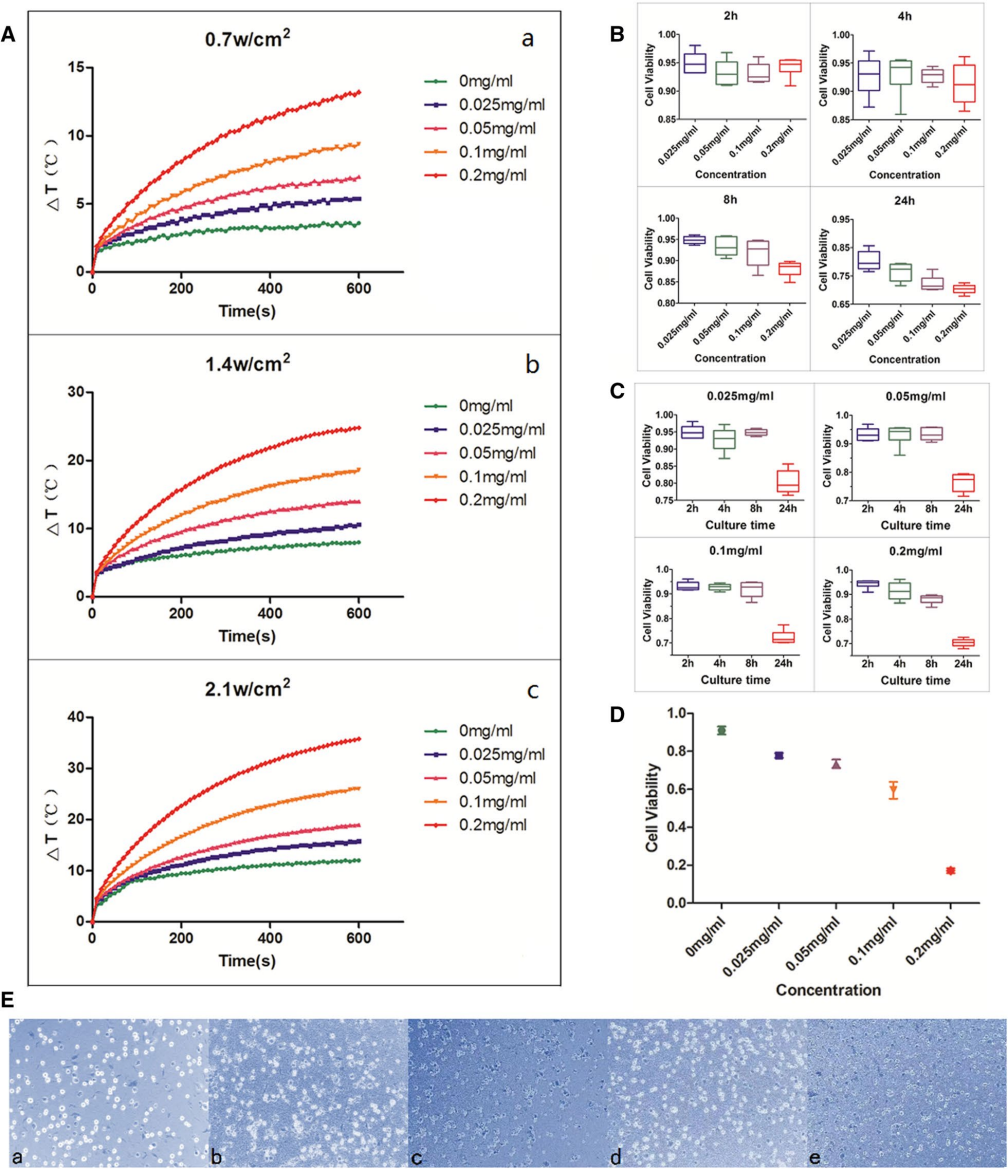
**A** The temperature changes of five different concentrations of USPIO-PEG-sLe^x^ nanoparticles under different power densities of NIR (a, b, and c) with a wavelength of 808 nm. **B** Cell viability of USPIO-PEG-sLe^x^ nanoparticles co-cultured with5-8F cells for 2 h, 4 h, 8 h, 24 h. **C** Cell viability of 5-8F cells in same concentration of USPIO-PEG-sLe^x^ nanoparticles at different culture times. **D** Cell viability of 5-8F cells with different concentrations USPIO-PEG-sLe^x^ nanoparticles in vitro PTT. **E** Trypan blue staining. Dead cells were stained blue, while living cells were translucent. The cell viability of each concentration was 80.00%, 75.21%, 78.33%, 75.80%, 69.45%, respectively (a, 0 mg/ml, b, 0.025 mg/ml, c, 0.05 mg/ml, d, 0.1 mg/ml, e, 0.2 mg/ml)

Through the photothermal property test of USPIO-PEG-sLe^x^ in vitro, our results demonstrated that the temperature of USPIO-PEG-sLe^x^ nanoparticles solution increased with its concentration and power density of NIR on 808 nm wavelengths. As the concentration increases, the temperature of USPIO-PEG-sLe^x^ nanoparticles solution increases significantly (Fig. 4A). The results show that USPIO-PEG-sLe^x^ nanoparticles have excellent photothermal conversion property in different concentrations of nanoparticles.

### Cytotoxicity of USPIO-PEG-sLe^x^

We tested the cytotoxicity of USPIO-PEG-sLe^x^ nanoparticles through Cell Counting Kit-8 (CCK8). Our results found that the viability of NPC 5-8F cells is related to the concentration of USPIO-PEG-sLe^x^ nanoparticles and the cultrue time (P < 0.001) (Table 1, Fig. 4B). When different concentrations of USPIO-PEG-sLe^x^ nanoparticles were co-cultured with 5-8F cells on 2 h and 4 h, viability of NPC 5-8F was no statistically significant difference between four concentration groups. However, when the co-culture time increased to 8 h, viability of NPC 5-8F between the 0.025 mg/ml concentration group and 0.1 mg/ml concentration group is statistically significant (P = 0.027), and 0.2 mg/ml concentration group a statistically significant difference from the other groups (P < 0.007), while the differences between the other groups are not statistically significant (P > 0.05). Cell viability was no difference between the concentrations of 0.1 mg/ml and 0.2 mg/ml at 24 h co-cultivation (P = 0.242), the differences between the other groups were statistically significant (P < 0.05). In addition, we analyzed the cell viability of 5-8F cells co-cultured with the same concentration of USPIO-PEG-sLe^x^ at different times. We found the cell viability differences between the 24 h time point and the other time points are statistically significant in the 0.025 mg/ml, 0.05 mg/ml and 0.1 mg/ml concentrations of USPIO-PEG-sLe^x^ (P < 0.001), and the differences are not statistically significant between the other time points (P > 0.05). When the concentration of nanoparticles is 0.2 mg/ml, the difference in cell viability at each time point is statistically significant (P < 0.039) (Fig. 4C). Therefore, we conclude that USPIO-PEG-sLe^x^ nanoparticles have slight cytotoxicity, but the increase in culture time and nanoparticle concentration will increase their toxicity.

**Table 1:** Cell viability of 5-8F cells co-cultured with different concentrations of USPIO-PEG-sLe^x^ nanoparticles at different time points

Concentration	n	Cell Viability			
		2 h	4 h	8 h	24 h
0.025 mg/ml	6	95.00% ± 1.92%	92.75% ± 3.37%	94.83% ± 0.91%	80.32% ± 3.39%
0.05 mg/ml	6	93.25% ± 2.35%	93.07% ± 3.62%	93.27% ± 2.12%	76.42% ± 3.12%
0.1 mg/ml	6	93.09% ± 1.76%	92.77% ± 1.29%	91.86% ± 3.18%	72.29% ± 2.74%
0.2 mg/ml	6	94.26% ± 1.72%	91.31% ± 3.55%	88.10% ± 1.78%	70.34% ± 1.64%

### In vitro PTT of USPIO-PEG-sLe^x^

The results of PTT in vitro indicated that the viability of 5-8F cells decreased significantly with the concentration of USPIO-PEG-sLe^x^ nanoparticles increases (P < 0.001) (Table 2, Fig. 4D), when USPIO-PEG-sLe^x^ nanoparticles underwent the 1.4 W/cm^2^ NIR power density and irradiated for 10 min. There was no statistically significant difference between the concentrations of 0.025 mg/ml and 0.05 mg/ml group (P = 0.242). The differences between the other groups were statistically significant (P < 0.001). These results meant that the high the concentration of nanoparticles had good PTT effect in vitro in the safe concentration range. Trypan blue cell staining also showed that the cell viability of 5-8F cells decreased with the increase of nanoparticles concentration (Fig. 4E).

**Table 2:** Cell viability of 5-8F cells with different concentrations USPIO-PEG-sLe^x^ nanoparticles in vitro PTT

Concentration	n	Cell Viability	F Value	P value
0 mg/ml	6	91.04% ± 5.20%	122.917	P < 0.001
0.025 mg/ml	6	77.83% ± 3.01%		
0.05 mg/ml	6	73.48% ± 5.55%		
0.1 mg/ml	6	59.50% ± 10.98%		
0.2 mg/ml	6	17.11% ± 3.14%		

### MRI of USPIO-PEG-sLe^x^ and USPIO-PEG, Prussian blue iron staining, and IHC staining

**Fig. 5:**
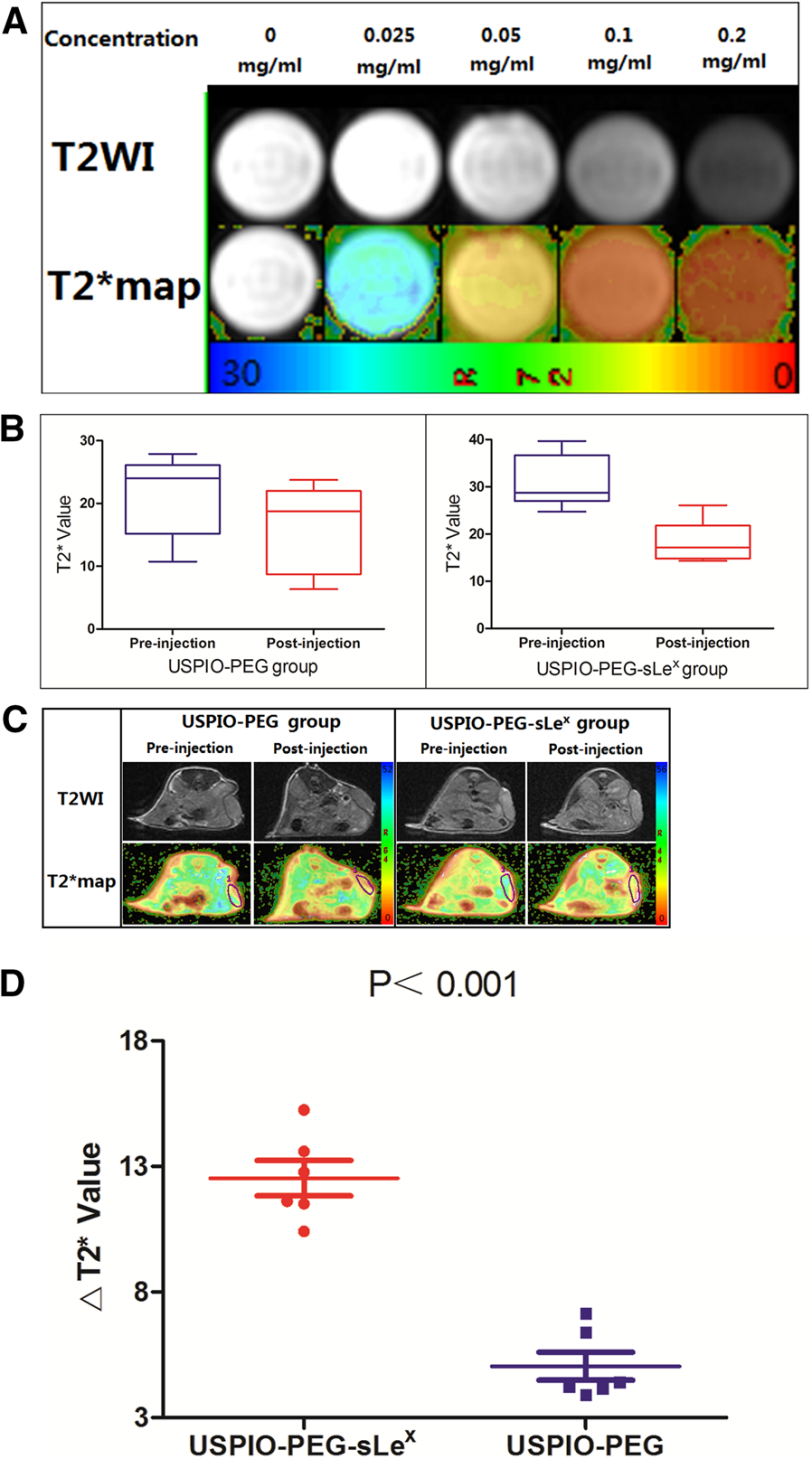
**A** T2WI signal changes of USPIO-PEG-sLe^x^ nanoparticles in different concentrations in vitro MRI. **B** Changes in tumor T2* values of tumor-burdened nude mice pre- and post-injection of nanoparticles in different groups. **C** There was a slight decrease in the signal visible to the naked eye from the tumor tissue via T2*map in the USPIO-PEG-sLe^x^ group, but there was not evident in the USPIO-PEG group. **D** TheΔT2* value of the USPIO-PEG-sLe^x^ group and the USPIO-PEG group

Our results showed that in vitro MRI, different concentrations of USPIO-PEG-sLe^x^ nanoparticles have a higher concentration of visible signal changes to the naked eye, increase the transverse relaxation rate, shorten the T2 relaxation time and reduce the T2 value (Fig. 5A).

In the USPIO-PEG-sLe^x^ group, compared with the tumor T2* value pre-injection of USPIO-PEG-sLe^x^ nanoparticles in vivo MRI, the tumor T2* value of tumor-burdened nude mice post-injection was lower, the T2* values was 30.870 ± 5.604 and 18.335 ± 4.351, respectively (Fig. 5B, C), the difference was statistically significant (P < 0.001). In the USPIO-PEG group, T2* values of pre- and post-injection was 21.465 ± 6.509 and 16.419 ± 6.910, respectively (Fig. 5B, C), the difference was statistically significant (P < 0.001).

In addition, we analyzed the T2* difference value (ΔT2* value) between the two groups pre- and post-injection. In the USPIO-PEG-sLe^x^ group and the USPIO-PEG group, theΔT2* value were 12.535 ± 1.730 and 5.046 ± 1.366, respectively. The difference was statistically significant (P < 0.001) (Fig. 5D). The results showed that both USPIO-PEG-sLe^x^ nanoparticles and USPIO-PEG nanoparticles reduced the T2* value in tumor area, especially USPIO-PEG-sLe^x^ nanoparticles. This means that USPIO-PEG-sLe^x^ nanoparticles has a targeted property. Moreover, USPIO-PEG nanoparticles can also reduce the T2* value in tumor area, and we inferred that the USPIO-PEG nanoparticles reached to tumor tissue through the tumor blood vessels.

**Fig. 6:**
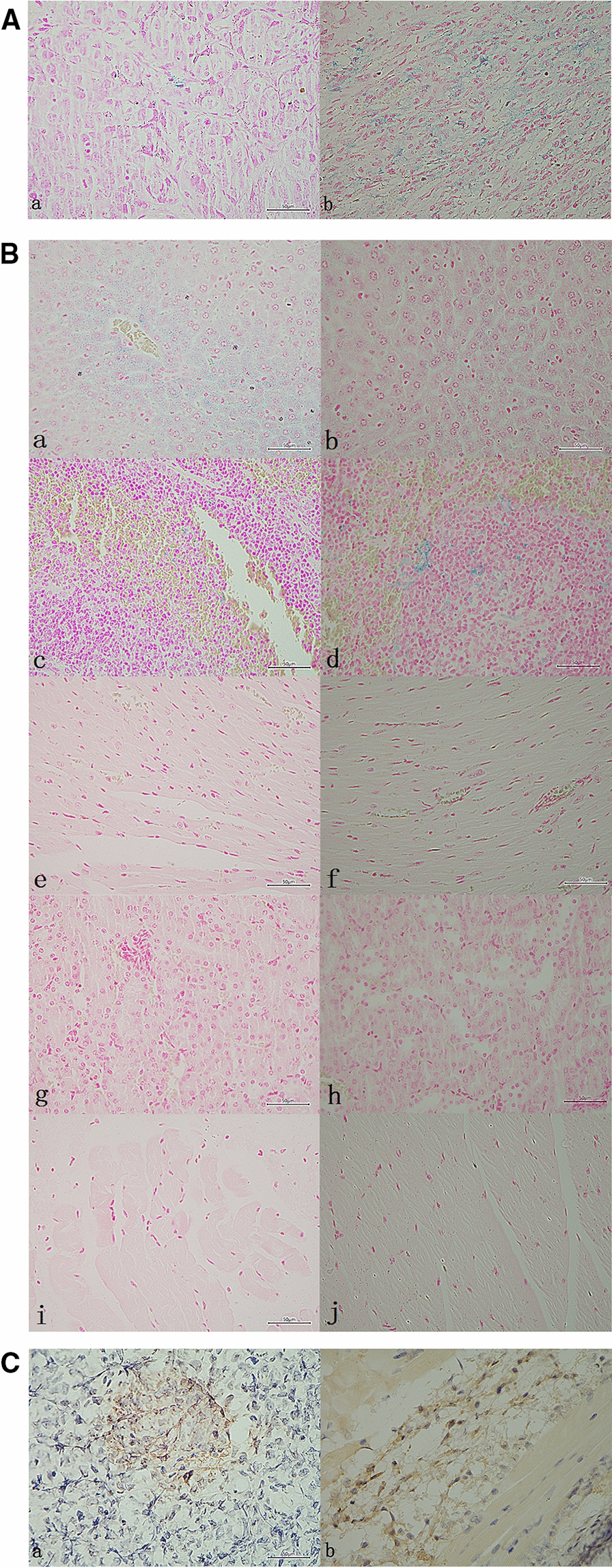
**A** Prussian blue iron staining of tumor specimens. (a, USPIO-PEG group, and there was a small amount of iron particles in the tumor tissue. b, USPIO-PEG-sLe^x^ group, and the blue reaction products were diffuse in the tumor.) **B** Prussian blue iron staining of different tissues. In A and B, The blue particles were iron particles and the red backgroud were tumor tissue (*A*) and normal organ tissue (**B**) (a, c, e, g, and i are liver, spleen, heart, kidney and muscle of nude mice in the USPIO-PEG group, respectively. And b, d, f, h, and j are liver, spleen, heart, kidney and muscle of nude mice in the USPIO-PEG-sLe^x^ group). **C** IHC staining. The brown area was the antigen–antibody reaction product, and the blue area was the nucleus. The expression of E-selectin was low positive in the both group (a, USPIO-PEG group and b, USPIO-PEG-sLe^x^ group). And it could be seen both cytoplasm and membrane

We performed the Prussian blue iron staining on the tumor specimens. We found that a few iron nanoparticles were stained blue in the tumor tissue in the USPIO-PEG group (Fig. 6Aa). By contrast, the amount of iron in the USPIO-PEG-sLe^x^ group was statistically more than that of the USPIO-PEG group (Fig. 6Ab). The results further explain why the decrease in T2* value of tumor tissue in the USPIO-PEG group after post-injection was not as significant as that in the USPIO-PEG-sLe^x^ group. We believe that USPIO-PEG-sLe^x^ nanoparticles can target the tumor area and can be used as a T2 contrast agent to perform MRI on nude mouse xenograft tumor models.

Prussian blue iron staining of hearts, livers, kidneys, spleens, and muscle were shown in Fig. 6B. The results showed that in both the USPIO-PEG-sLe^x^ group and the USPIO-PEG group, the nanoparticles concentrated in the liver, and there was also a small amount of iron in the spleens, but hardly any in other organs. This implied that the targeted component sLe^x^ has no effect on the biodistribution of USPIO-PEG, and iron is mainly metabolized through the liver in mice.

IHC analysis showed brown reaction products of tumors specimens in two groups, but the expression level was low (Fig. 6C). The IHC results of tumor specimens in both groups were low positive via ImagJ software annalysis. It indicated that E-selectin of xenograft model was a low expression state.

### PTT of USPIO-PEG-sLe^x^ in vivo

**Fig. 7:**
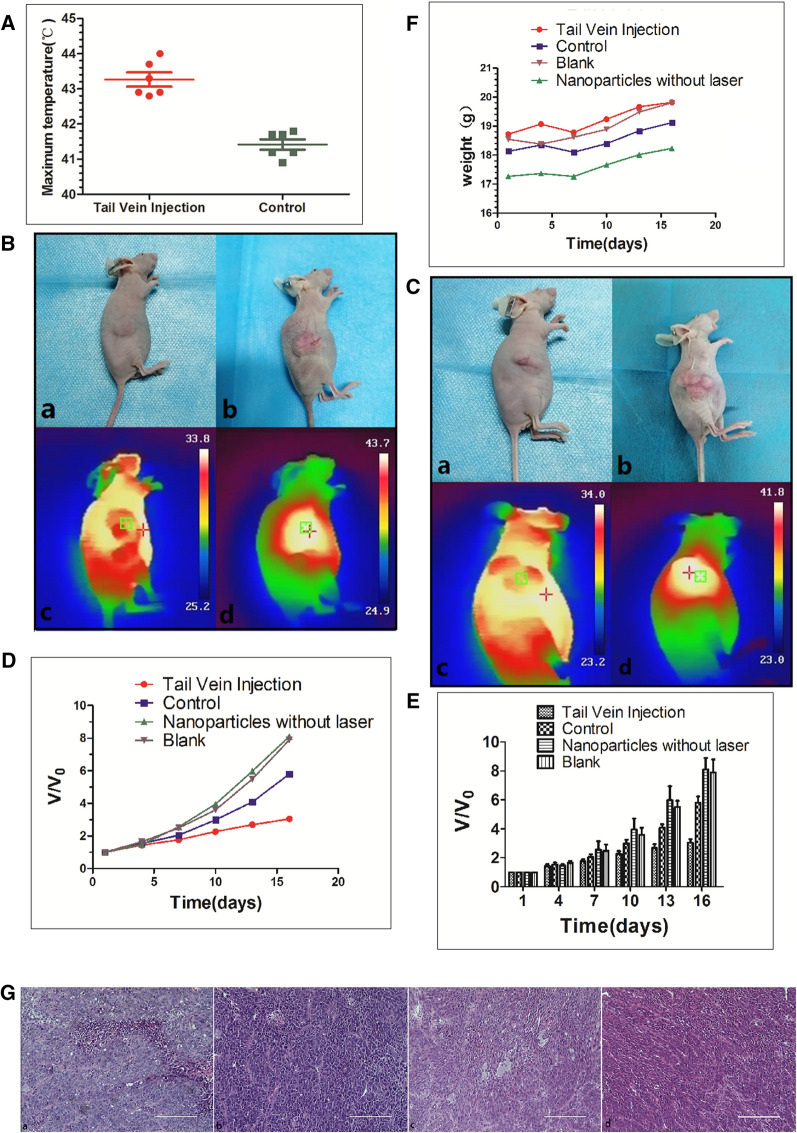
**A** The highest temperature of nude mice xenografts in different groups after 10 min of NIR light irradiation. **B** Tail vein injection group. "+" is the highest temperature area which measured by NIR imager. (a) Tumor before treatment, (b) Tumor after 5 treatments, (c) The maximum temperature is 33.8 °C (non-tumor tissue temperature) before treatment, (d) The maximum temperature after 10 min of treatment (tumor tissue temperature) reached 43.7 °C. **C** Control group. "+" is the highest temperature area which measured by NIR imager. (a) Tumor before treatment, (b) Tumor after treatment 5 times, (c) The highest temperature before treatment is 34.0 °C (non-tumor tissue temperature), (d) The highest temperature after treatment reaches 41.8 °C 10 min (tumor tissue temperature). **D** and **E** Changes in tumor volume pre- and post-treatment in the tail vein injection group, control group, nanoparticles without laser irradiation group and blank group. **F** Changes of weight in nude mice pre- and post-treatment among the tail vein injection group, control group, nanoparticles without laser irradiation group and blank group. **G** HE staining. More apoptotic cells were seen in the tail vein injection group (a), but very few in the control group (b), the blank group (c) and the nanoparticles without laser irradiation group (d)

The real-time temperature of PTT treatment in the tail vein injection group and the control group was monitored with NIR imager. Our results showed that after 10 min of NIR light irradiation, the temperature of the tumor tissue in the tail vein injection group was higher than that in the control group (Table 3, Fig. 7A), and the difference was statistically significant (P < 0.001). The result showed that the tail vein injection group had USPIO-PEG-sLe^x^ at the tumor site. This is consistent with the results of in vivo MRI experimental studies.

**Table 3:** Comparison of the highest temperature in the two groups of nude mice xenografts after 10 min of NIR light irradiation

Group	n	Maximum temperature (°C)	t Value	P value
Tail vein injection	6	43.267 ± 0.493	7.387	P < 0.001
Control	6	41.417 ± 0.366

**Fig. 8:**
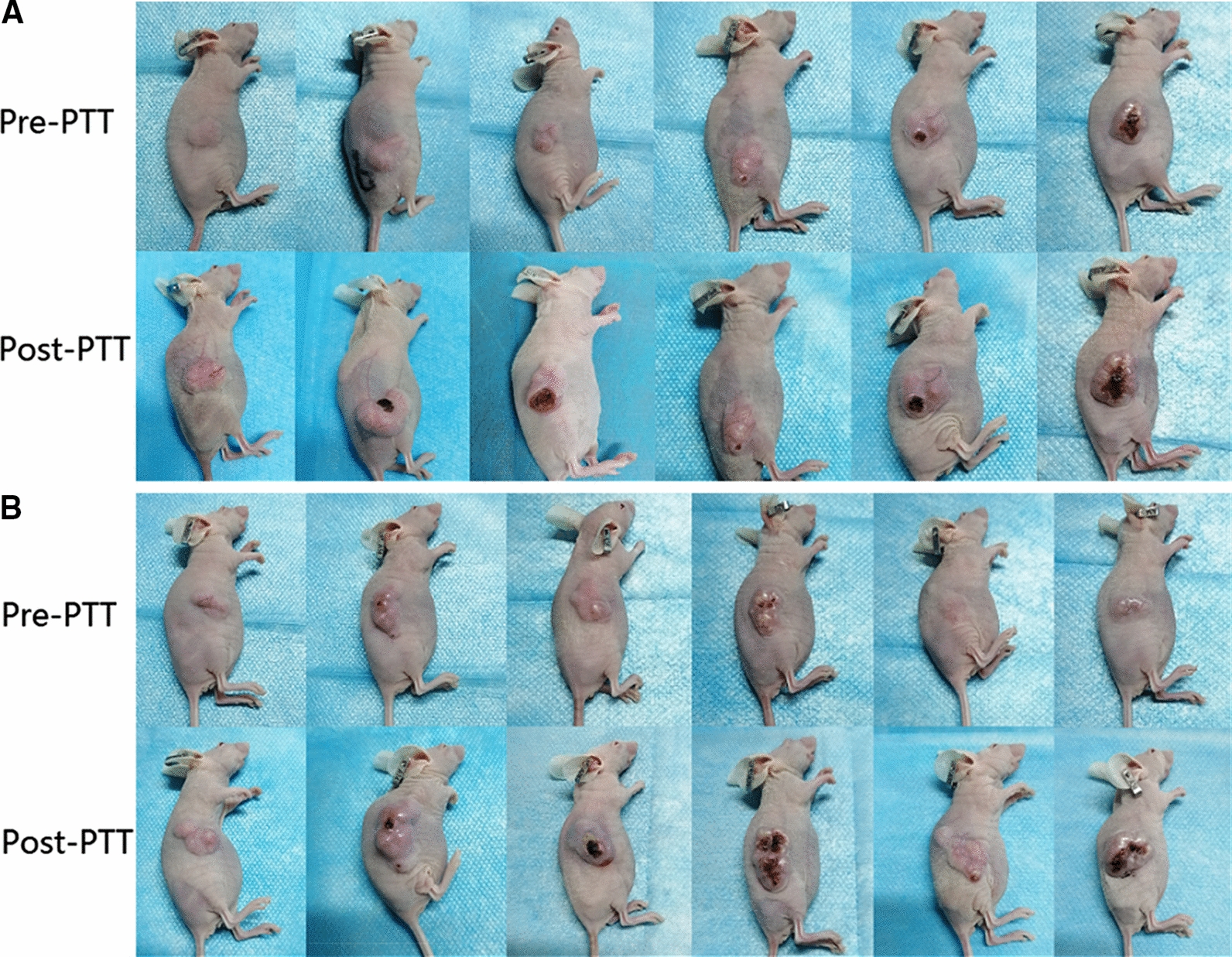
**A** Tail vein injection group. **B** Control group. The top row were the tumor pre-treatment, and the bottom row were the tumor after five PTT

We recorded the temperature changes in the tumor tissue and surrounding tissue pre- and post-treatment in the tail vein injection group and the control group (Fig. 7B, C). Changes of tumor pre- and post-treatment were also recorded (Fig. 8). Volume measurement in the xenograft tumor between the tail vein injection group, the control group, the nanoparticles without laser irradiation group and the blank group. Our results indicate the ratio of the volume change between tail vein injection group, control group, nanoparticles without laser irradiation group and blank group after 5 treatments was 3.04 ± 0.57, 5.80 ± 1.06, 8.09 ± 1.96, 7.89 ± 2.20, respectively (Fig. 7D, 7E). The difference between these four groups are statistically significant (P < 0.001). Pairwise comparisons between nanoparticles without laser irradiation group and blank group was no statistically significant difference (P = 0.828). The differences among other groups were statistically significant (tail vein injection group vs control group P = 0.007, tail vein injection group vs nanoparticles without laser irradiation group P < 0.001, tail vein injection group vs blank group P < 0.001, control group vs nanoparticles without laser irradiation group P = 0.021, control group vs blank group P = 0.034, respectively). During the experiment period, no tumor-burdened nude mice died, and the average weight of tumor-burdened nude mice in all four groups increased (Fig. 7F), which indirectly indicated that the USPIO-PEG-sLe^x^ nanoparticles has no obvious biological toxicity and will not cause cachexia in the short term. Therefore, we believe that USPIO-PEG-sLe^x^ nanoparticles can significantly inhibit tumor progression and can serve as a nanothermal platform for PTT.

### HE staining

Our HE staining results found that more apoptotic cells detected in the tumor specimens of the tail vein injection group in the PTT in vivo, but not in the control group, nanoparticles without laser irradiation group and the blank groups (Fig. 7G). Compared with the other groups, the tumor progression in the tail vein injection group was significantly inhibited. The PTT caused more tumor cells apoptosis, but PTT did not kill the tumor cells. We speculated that the E-selectin was a low expression state on the surface of vascular endothelial cells for the absence of metastasis or weak invasion in our xenograft model. It resulted in a low concentration of USPIO-PEG-sLe^x^ nanoparticles targeting the tumor site, and failed to cause obvious apoptosis in tumor cells. The results of IHC staining can also verify our inference.

## Discussion

We have prepared a nanotheranostic platform for MRI and PTT in human NPC xenografts on mice model. We have confirmed that the nanotheranostic platform have two functions for MRI and PTT, and can predominately inhibit xenografts tumor progression in vivo and in vitro experiment. Although there were several similar systems that could be used for MRI and PTT, our synthesized nanoparticles had some advantages. E-selectin overexpression is associated with tumor metastasis, and studies have found that E-selectin expressed in a variety of tumor tissues, such as NPC [21]. Therefore, our nanotheranostic platform could be effectively applied in photothermal therapy and molecular imaging of a variety of tumors. In addition, the chemotherapy dilemma of local advanced NPC treatments were low selectivity and large side effects. Our nanotheranostic platform can accurately target the tumor region, improve the selectivity and have fewer side effects in NPC treatment.

PTT based on tumor blood vessels may remodel of the Cell–cell and cell-extracellular matrix which seems promising for decreasing mechanical stress in the tumor microenvironment, the degradation of such components may also have unwanted outcomes, such as promoting invasiveness and migration of cancer cells [27, 28]. In future, We need for improved understanding and careful design of targeted regimens, and PTT is not necessarily in one singular strategy, but rather the development of leverage double or multiple regimes, such as simple thermotherapy (hyperthermia) combined low-dose sunitinib (anti-angiogenic therapy) [29, 30].

At present, the bottleneck of NPC treatments are low selectivity and large side effects. However, the nanotheranostic platform we prepared can accurately target the tumor region, improve the selectivity of treatment, and have fewer side effects. It might break the bottleneck. PTT has become a new strategy for anti-tumor treatment due to its many advantages, but each treatment has its advantages and limitations. Although PTT can inhibit tumor growth and promote cell apoptosis, it is not feasible to use PTT alone for anti-tumor therapy and only be used as adjuvant therapy. And a lot of research has been done on PTT in combination with other strategies. How to improve the targeting property and photothermal conversion efficiency and control the therapeutic concentration of PAs in the target area is the key to successful treatment. In our study, one of the the limitations is that the concentration of USPIO-PEG-sLe^x^ nanoparticles in the tumor area is not high enough. If an additional external magnetic field is applied to the tumor area, it may be able to increase the concentration of USPIO-PEG-sLe^x^ nanoparticles in the tumor to improve the PTT effect because USPIO-PEG-sLe^x^ nanoparticles is a superparamagnetic iron oxide nanoparticle. Due to the heterogeneity of tumors, the concentration of nanoparticles in the xenografts of nude mice in the same experimental group also varied, which is another limitation of this study. The third limitation is that sLe^x^ theoretically targets vascular endothelial cells, however, we did not experiment on vascular endothelial cells. And the fourth limitation is that we did not test the heat stability of USPIO-PEG-sLe^x^ nanoparticles. In future, we will explore other protein molecules overexpressed of NPC cells. We do our best to improve the targeting property of the nanotheranostic platform in order to enhance the curative effect of PTT via couple specific ligands of sLe^x^ and other protein molecules in USPIO-PEG simultaneously. Further improving the targeting property of this nanotheranostic platform, it may be become a new strategy for the treatment of NPC.

## Conclusions

We synthesized the USPIO-PEG-sLe^x^ nanotheranostic platform, and it may break through the bottleneck of traditional therapies of NPC and become a new strategy for NPC treatment.

## Data Availability

All data generated or analysed during this study are included in this published article.

## Abbreviations

NPC: Nasopharyngeal carcinoma
USPIO: Ultrasmall superparamagnetic iron oxide
PEG: Polyethylene glycol
sLe^x^: Sialyl Lewis X
USPIO-PEG-sLe^x^: Polyethylene glycol-coated ultrasmall superparamagnetic iron oxide nanoparticles-coupled sialyl Lewis X
PTT: Photothermal therapy
MRI: Magnetic resonance imaging
IARC: The International Agency for Research on Cancer
PA: Photothermal agent
NIR: Near-infrared
FDA: The American Food and Drug Administration
TEM: Transmission electron microscopy
DLS: Dynamic light scattering
FTIR: Fourier Transform Infrared
CCK8: Cell Counting Kit-8
